# Reuterin Demonstrates Potent Antimicrobial Activity Against a Broad Panel of Human and Poultry Meat *Campylobacter* spp. Isolates

**DOI:** 10.3390/microorganisms8010078

**Published:** 2020-01-06

**Authors:** Paul Tetteh Asare, Katrin Zurfluh, Anna Greppi, Denise Lynch, Clarissa Schwab, Roger Stephan, Christophe Lacroix

**Affiliations:** 1 Laboratory of Food Biotechnology, Institute of Food, Nutrition and Health, ETH Zürich, 8092 Zürich, Switzerland; paul.asare@hest.ethz.ch (P.T.A.); anna.greppi@hest.ethz.ch (A.G.); clarissa.schwab@hest.ethz.ch (C.S.); 2 Institute for Food Hygiene and Safety, University of Zürich, 8057 Zürich, Switzerland; katrin.zurfluh@uzh.ch (K.Z.); denise.lynch@uzh.ch (D.L.); stephanr@fsafety.uzh.ch (R.S.)

**Keywords:** Acrolein, reuterin, antimicrobial, *Lactobacillus reuteri*, *Campylobacter*

## Abstract

Reuterin is a broad-spectrum antimicrobial system produced by specific strains of *Lactobacillus reuteri* during anaerobic metabolism of glycerol. Acrolein is the main component responsible for its antimicrobial activity. Here, the sensitivity of *Campylobacter jejuni* (*n* = 51) and *Campylobacter coli* (*n* = 20) isolates from chicken meat and human stool samples to reuterin was investigated. The minimum inhibitory concentration (MIC) of *C. jejuni* and *C. coli* strains was measured between 1.5 and 3.0 µM of acrolein, below the MIC of the sensitive indicator strain *Escherichia coli* K12 (16.5 µM acrolein). The interaction of *C. jejuni* N16-1419 and the reuterin-producing *L. reuteri* PTA5_F13 was studied during 24 h co-cultures with or without glycerol. A high *C. jejuni* growth was observed in cultures without glycerol. In contrast, *C. jejuni* growth decreased from 7.3 ± 0.1 log CFU/mL to below detection limit (1 log CFU/mL) during co-cultures added with 28 mM glycerol. This bactericidal effect could be attributed to in situ reuterin production. The low MIC observed and the high sensitivity towards in situ produced reuterin suggests *L. reuteri* combined with glycerol, as a possible intervention option to reduce *Campylobacter* in the food chain.

## 1. Introduction

*Campylobacter* spp., mainly *C. jejuni* and *C. coli*, are the most commonly reported foodborne pathogens in the European Union, with 246,307 confirmed cases of human campylobacteriosis in 2016 [1]. Related economic costs are estimated to be around 2.4 billion € in the EU per year [2]. Most *Campylobacter* infections occur as sporadic cases rather than as outbreaks [3]. Several epidemiological studies indicated that improper handling of raw meat from chickens that carry a high load of *Campylobacter* is the major source of human infections [4].

*Campylobacter* infections in humans are usually self-limiting and do not require antibiotic therapy [5]. However, in severe cases, antibiotics such as ciprofloxacin, tetracycline and erythromycin can be prescribed [6]. However, the efficiency of antibiotics against *Campylobacter* infections is decreasing due to an increase in antibiotic resistance [7]. The use of antibiotics in poultry production may contribute to the emergence of resistant strains in human through the food chain [8]. Nowadays, there is an increasing interest in intervention strategies to reduce the presence of *Campylobacter* spp. in the poultry meat production chain to lower the risk of *Campylobacter* exposure.

**Figure 1:**
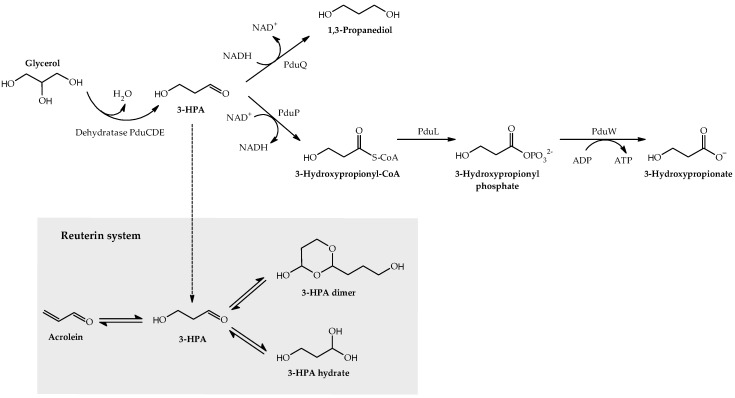
Glycerol metabolism by reuterin-producing *L. reuteri*. Anaerobic metabolism of glycerol by reuterin producing *L. reuteri* to 3-hydroxypropionaldehyde (3-HPA) and further to 3-hydroxypropionate (3-HP) and 1,3-propanediol (1,3-PDO). In an aqueous environment, 3-HPA is quickly dimerised and hydrated to form HPA-dimer and HPA-hydrate and also spontaneously dehydrates to acrolein. PduQ, 1,3-PDO dehydrogenase; PduP, CoA-dependent propionaldehyde dehydrogenase; PduL, phosphotransacetylase; PduW, propionate kinase.

Reuterin is a potent antimicrobial system produced by certain strains of *Lactobacillus reuteri* from glycerol in a single reaction catalysed by the enzyme glycerol/diol dehydratase PduCDE [9]. Reuterin is a dynamic multi-compound system consisting of 3-hydroxypropionaldehyde (3-HPA), 3-HPA hydrate, 3-HPA dimer and acrolein [10]. 3-HPA can be further metabolised to 1,3-propanediol (1,3-PDO) and 3-hydroxypropionate (3-HP) by the enzymes encoded by the propanediol-utilisation (*pdu*) operon (Figure 1). Several studies found that reuterin solutions exhibit antimicrobial activities against a broad range of Gram-positive and Gram-negative bacteria, yeasts, moulds, and protozoa [11]. This activity has been attributed to reuterin causing depletion of free thiol groups in glutathione (GSH), proteins, and enzymes, resulting in an imbalance of the cellular redox status which leads to bacterial cell death [12]. We recently showed that acrolein is the main component responsible for the antimicrobial activity of reuterin [10,13].

*L. reuteri* is known to form stable biofilms in the crop and to persist in poultry gastrointestinal (GI) tracts [14]. In the poultry industry, glycerol is used as energy feedstuff [15] and to improve feed pellet quality [16]. Therefore, in situ reuterin production may be used as an active natural mechanism in chicken colonised with reuterin-producing *L. reuteri*, to inhibit enteropathogens such as *Campylobacter* in the gastrointestinal tract (GIT). However, to our knowledge, there is limited information on the sensitivity of *Campylobacter* spp. to this broad-spectrum antimicrobial system. In this study, we assessed the potential of reuterin to inhibit *Campylobacter* spp. The antimicrobial efficacy of reuterin was evaluated on a panel of *C. jejuni* and *C. coli* isolated from various sources, including human stools, chicken GIT and meat. The bactericidal activity of *in situ*-produced reuterin on *C. jejuni* was tested in co-cultures with reuterin-producing *L. reuteri*, in the presence and absence of glycerol.

## 2. Materials and Methods

### 2.1. Bacterial Strains, Media and Growth Conditions

Seventy-one (71) *Campylobacter* spp. (*C. jejuni* (*n* = 51) and *C. coli* (*n* = 20)) isolated from the chicken intestine (*n* = 2), human stool samples (*n* = 50) and chicken meat (*n* = 19) in 2016 and 2017 with different antibiotic resistance profiles were supplied by the National Centre for Enteropathogenic Bacteria and Listeria (NENT; University of Zurich, Zurich, Switzerland) (Table S1). Before use, *Campylobacter* strains were sub-cultured twice at 41 °C for 48 h on Blood Agar, 5% sheep blood (Oxoid AG, Pratteln, Switzerland) under microaerobic conditions (5% O_2_, 10% CO_2_, 85% N_2_) generated using gas package (Campygen, Oxoid AG, Pratteln, Switzerland). Cation adjusted Mueller Hinton broth (CAMHB) (Becton Dickinson AG, Allschwil, Switzerland) was used for routine cultivation of *C. jejuni* and *C. coli*. Quantification of *C. jejuni* was performed using CampyFood ID Agar (BioMérieux, Geneva, Switzerland).

*L. reuteri* DSM20016 was obtained from the DSM strain collection (Leibniz Institute DSMZ-German Collection of Microorganisms and Cell Cultures, Braunschweig, Germany). *L. reuteri* PTA8_11 (non-reuterin producer) and *L. reuteri* PTA5_F13, PTA6_C2 and PTA4_C2 (all three reuterin-producers) were isolated from poultry gut and shown to share average nucleotide identity (ANI) of 95% with reference strain *L. reuteri* DSM20016T [17]. They were obtained from our culture collection (Laboratory of Food Biotechnology, ETH-Zürich, Switzerland). The reuterin producer chicken strains share sequence similarity (98%) with the non-reuterin producer strain, while the latter lacks the glycerol/diol dehydratase PduCDE (EC 4.2.1.30) required for reuterin synthesis [9]. These strains phylogenetically cluster together in the poultry/human VI clade of *L. reuteri*, previously described [18]. Details on the isolation, characterisation and efficiency of reuterin production of these strains were reported by Greppi et al. [17].

*L. reuteri* strains were routinely cultivated in Man, Rogosa and Sharpe medium (MRS, Biolife, Milan, Italy) at 37 °C under anaerobic condition supplied by gas package (AnaeroGen, Thermo Fisher Diagnostics AG, Pratteln, Switzerland). The reuterin activity indicator strain *E. coli* K12 (ER2925) (New England Biolabs, Ipswich, MA, USA) was cultured in brain heart infusion medium (BHI, Biolife, Milan, Italy), at 37 °C. All bacterial strains were sub-cultured three times in liquid suspension for 14 h at 37 °C before use.

### 2.2. Reuterin Production

*L. reuteri* PTA5_F13 was used for reuterin production with a two-step process using 600 mM glycerol, as previously described [13]. Briefly, cell pellets obtained from exponentially grown *L. reuteri* PTA5_F13 were suspended in 600 mM glycerol solution and incubated at 25 °C for 2 h. The concentrations of 3-HPA and acrolein of the reuterin solution (supernatant) were measured by high-performance liquid chromatography with refractive index detector (HLPC-RI) and ion-exclusion chromatography with pulsed-amperometric detection (IC-PAD) analysis.

### 2.3. Reuterin Antimicrobial Activity Against a Panel of C. jejuni and C. coli Strains

The minimal inhibitory concentrations (MIC) and the minimal bactericidal concentrations (MBC) of reuterin were determined using a broth microdilution assay, as previously described [19] with some modifications. For antimicrobial activity testing, a fresh working solution of reuterin containing 1.4 mM acrolein was prepared in CAMH broth. Briefly, inocula were prepared from overnight grown *C. jejuni* and *C. coli* on blood agar plates by resuspending colonies in sterile saline to obtain a turbidity equivalent to 0.5 McFarland standard, corresponding to 5 × 10^5^ colony forming units (CFU) per mL [20]. A hundred microliters of the working reuterin solution was added in the first row of a 96-well microtiter plate (tissue culture plate with a flat bottom, Bioswisstec AG, Schaffhausen, Switzerland). In each of the remaining wells, 50 µL CAMHB was added. Then a serial two-fold dilution was done by pipetting 50 µL from column 1 to 11. Reuterin was not added to the last well of a column which was used as positive growth control. Each well was then inoculated with the 0.5 McFarland-standardized bacterial suspension. *L. reuteri* strains and *E. coli* K12 cells were tested in parallel and used as indicator organisms for low and high sensitivity control of reuterin, respectively [21]. Both cultures were prepared in their respective medium, and the inoculum was adjusted to 0.5 McFarland standard, as presented above. The microtiter plates were incubated at 41 °C under microaerophilic and static conditions for 24 h. The OD600 was measured using a PowerWave XS microplate spectrophotometer (BioTek, Sursee, Switzerland). The MIC was defined as the first (lowest) dilution showing growth-inhibition (OD600 < 0.1). The MBC was determined by spotting 10 µL of the dilution well corresponding to MIC, as well as two-fold dilutions on CampyFood ID Agar. Agar plates were incubated in microaerophilic conditions for 48 h in jars. The MBC was defined as the lowest dilution at which no growth on the agar plate was observed. The MIC and MBC of reuterin against *Campylobacter* were expressed in µM acrolein, since acrolein has been shown to be the main active component of the reuterin system [10,13]. Each strain was tested three times independently, and the results expressed as ranges of MIC or MBC in µM of acrolein.

### 2.4. Glycerol Metabolism by Mono-Culture Assay of L. reuteri and C. jejuni under Microaerophilic Growth Condition

Glycerol is the main substrate required for reuterin synthesis by *L. reuteri*. We tested the ability of non-reuterin producing (*L. reuteri* PTA8_1) and reuterin-producing *(L. reuteri* PTA5_F13) to synthesise reuterin from glycerol under the microaerophilic conditions required for optimal growth of *Campylobacter* spp. We also measured the effect of glycerol supplementation on the growth and metabolism of a reuterin-sensitive strain, *C. jejuni* N16-1419.

*L. reuteri* PTA8_1 and *L. reuteri* 5_F13 were grown overnight (16 h) in MRS broth at 37 °C. *C. jejuni* N16-1419 was incubated for 24 h in CAMH broth at 41 °C under microaerobic conditions. Cells were harvested at 3000× *g* for 2 min, washed once with phosphate buffer saline (PBS) and resuspended with PBS to an OD600 of 6.0 (5.0 × 10^9^ CFU/mL) for *L. reuteri* and 7.0 (1.0 × 10^10^ CFU/mL) for *C. jejuni*. The cultures (300 µL) were inoculated at 1% (*v*/*v*) into 30 mL of CAMH broth containing 28 mM glycerol. The inoculated medium was dispensed on 30 mL CAMH agar containing 28 mM glycerol in 75-cm^2^ tissue culture flasks with vented cap (Sigma-Aldrich, Buchs, Switzerland), forming a bi-phasic medium previously recommended for optimal growth of *Campylobacter* spp. [22]. Inoculated bi-phasic CAMH broth and agar without glycerol were used as control. Flasks were then incubated horizontally at 41 °C, under microaerophilic conditions for 24 h. Cell growth was quantified by OD600, qPCR and plate count after 24 h incubation. Metabolite production was measured in the culture supernatant using HPLC-IR and IC-PAD. For viable cell counts determination, a ten-fold dilution series of 100 µL samples of each culture were plated on CampyFood ID agar and MRS agar for the enumeration of *C. jejuni* and *L. reuteri*, respectively. Plates for *C. jejuni* quantification were incubated in jars under microaerophilic conditions generated by gas package (CampyGen, Thermo Fisher Diagnostics AG, Pratteln, Switzerland) at 41 °C for 24 h. Plates for *L. reuteri* were incubated under anaerobic condition supplied by gas package (AnaeroGen, Thermo Fisher Diagnostics AG, Pratteln, Switzerland) overnight for 37 °C. Three independent replicates were performed.

### 2.5. Co-Culture Assays of L. reuteri and C. jejuni with Glycerol

Based on the results derived from microaerophilic growth of *L. reuteri* PTA8_1 and PTA5_F13 and *C. jejuni* 16-1419 in CAMH broth with or without glycerol, we designed a co-culture assay to assess the effect of in situ production of reuterin by *L. reuteri* on the survival and growth of *C. jejuni*.

For co-culture experiments, an inoculum of *L. reuteri* PTA8_1 or PTA5_F13 cultures prepared as described above were co-inoculated with *C. jejuni* N16-1419 (all at 1%) in CAMH broth (30 mL) containing 28 mM glycerol. The incubation of the co-cultures were similar as described for mono-cultures. The abundance of *C. jejuni* N16-1419 in co-culture was determined after 24 h incubation using viable plate counts on selective media (CampyFood ID Agar) and 16S rRNA gene quantitative PCR. The pH and metabolites were measured. Three independent replicates were performed.

### 2.6. DNA Isolation and Quantitative PCR (qPCR) Analysis

Total genomic DNA was extracted from 2 mL samples of the mono-cultures and co-cultures using the Fast DNA SPIN kit for soil (MP Biomedicals, Illkirch, France), following manufacturer’s instructions. The DNA concentration and quality were assessed by absorbance measurements at 260 nm on a NanoDropVRND-1000 Spectro-photometer (Witec AG, Littau, Switzerland), and samples were stored at −20 °C prior to the qPCR analyses.

To amplify and quantify the 16S rRNA gene copies, the primers specific for the 16S rRNA gene for *Lactobacillus* (FWD: 5′-AGC AGT AGG GAA TCT TCC A-3′, REV: 5′-CGC CAC TGG TGY TCC ATA TA-3′) [23] and *Campylobacter* (FWD: 5′-CTG CTT AAC ACA AGT TGA GTA GG-3′, REV: 5′-TTC CTT AGG TAC CGT CAG AA-3′) [24] were used. Total DNA (1 µL) was used for amplification in duplicate in 20 µL total reaction solution, containing 10 μL of SensiFAST SYBR No-ROX Kit (Bioline, Luckenwalde, Germany) and 10 pmol of each primer. qPCR reactions were performed using a Roche LightCycler 480 II, (Roche Diagnostics AG, Rotkreuz, Switzerland) in multiwell plate 96 at 95 °C for 3 min, followed by 45 cycles at 95 °C for 5 s, 60 °C for 30 s. At the end of the qPCR cycles, melting curve analysis was performed to validate the specific generation of the expected PCR products. Each reaction was run in duplicate. For quantification, a dilution series of standard obtained by amplification of the linearised plasmid containing the representative gene of the target bacterial species was included in each run. qPCR data were analysed using the LightCycler^®^ 480 Software 1.5.1 (Roche Diagnostics AG, Rotkreuz, Switzerland). PCR efficiency (%) was calculated from the slope of the standard curve for each qPCR assay. Assay with an efficiency of 80–110% (slope 3.2–3.9) were retained. The number of 16S rRNA gene copies measured were corrected for multiple copies to estimate the number of *C. jejuni* (2.1 mean gene copies) and *L. reuteri* (6.0 mean gene copies) cells using rrnDB version 5.5 [25].

### 2.7. Chemical Analyses

Glycerol, 1,3-PDO, 3-HPA, and microbial fermentation metabolites such succinate, acetate, propionate and butyrate concentrations were determined by HPLC with refractive index detector (HPLC-RI, Hitachi LaChrome, Merck, Dietikon, Switzerland) on an Aminex HPX-87H column (300 × 7.8 mm, Bio-Rad, Reinach, Switzerland), as previously described [13]. Purification of 3-HPA used as a standard was carried out as previously described [26].

Acrolein and 3-HP concentration were determined using ion-exclusion chromatography with pulsed amperometric detection (IC-PAD) [10]. Commercial pure acrolein (>99%, stabilised with 0.2% hydroquinone) from Sigma-Aldrich GmbH (Buchs, Switzerland) was used as an external standard. Due to the overlapping of glycerol and lactate peaks in HPLC-RI chromatograms, total lactate in the culture samples was enzymatically measured using D-/L-lactate (Rapid) Assay Kit (Megazyme, Wicklow, Ireland), according to the manufacturers’ instruction.

### 2.8. Statistical Analysis

Data are mean values with a standard deviation of biological replicates. Statistical comparison of metabolite productions, viable cell counts and qPCR data of mono-cultures and co-cultures with or without 28 mM glycerol was evaluated by Students t-test using IBM SPSS 24.0 (IBM SPSS Statistics for Windows, NY, USA). Significance was set at *P*-value of less than 0.05 (two-tailed).

## 3. Results

### 3.1. C. jejuni and C. coli Strains are Highly Sensitive to Reuterin Compared to E. coli K12 Indicator

The antimicrobial activity of reuterin expressed in acrolein concentration was tested against a panel of 71 strains of *C. jejuni* and *C. coli* isolated from human stools (50) and chicken GIT (19) and meat (2). The reuterin stock solution was produced using *L. reuteri* PTA5_F13 and contained 402.9 mM 3-HPA, 51.2 mM 1.3-PDO and 8.5 mM acrolein from 600 mM glycerol. All tested *Campylobacter* strains exhibited similar high sensitivity to reuterin with very low MIC and MBC in the range of 1.5 to 5.8 µM, which were approximately ten folds lower than for *E. coli* K12 (Table 1). As expected, reuterin-producing *L. reuteri* isolated from chicken showed lower sensitivity to reuterin, with MIC and MBC approximately tenfold and hundredfold higher than *E. coli* K12 and *Campylobacter* strains, respectively.

### 3.2. *L. reuteri* PTA5_F13 Produces Reuterin under Microaerophilic Growth Conditions

Prior to co-culture trials, the production of reuterin of the selected strain, *L. reuteri* PTA5_F13, was tested in the microaerophilic conditions used for *Campylobacter* growth, and the effect of glycerol was as tested during *Campylobacter* mono-cultures.

There was no significant difference (*p* > 0.05) in abundance (colony counts and 16S rRNA gene copies) and metabolic activity of *C. jejuni* N16-1419 in bi-phasic CAMH medium with or without 28 mM glycerol after 24 h incubation (Table 2 and Table 3). *C. jejuni* N16-1419 viable cell counts reached 8 log CFU/mL, and succinate (6 mM) and acetate (4 mM) were the main metabolites produced. However, the pH remained similar to the initial pH of the well-buffered CAMH medium (Table 2).

The non-reuterin producing *L. reuteri* PTA8_1 did not grow under the microaerophilic condition. Glycerol was not consumed, and there was no significant difference (*p* > 0.05) in abundance and metabolite concentrations after 24 h incubation in bi-phasic CAMH medium with or without 28 mM glycerol (Table 2 and Table 3). In these conditions, *L. reuteri* PTA8_1 produced acetate and lactate, and the pH decreased from 7.3 for the non-inoculated CAMH medium to 6.8 after 24 h incubation (Table 2).

Similar to non-reuterin producing *L. reuteri* PTA8_1, we did not observe growth of the reuterin-producing *L. reuteri* PTA5_F13 after 24 h incubation in CAMH medium under microaerophilic condition for 24 h. However, *L. reuteri* PTA5_F13 converted approximately 13 mM (54%) glycerol to produce 0.2 ± 0.0 mM acrolein, 5.3 ± 0.3 mM 1,3-PDO, and 4.4 ± 0.3 3-HP (Table 2). Besides, significantly (*p* < 0.05) higher levels of acetate and lactate were produced in the presence of glycerol compared to without. A significantly lower (*p* < 0.05) viable cell counts was measured after 24 h incubation with glycerol (4.5 ± 0.4 CFU/mL) compare to without glycerol (6.7 ± 0.1 CFU/mL). However, no difference (*p* > 0.05) was observed for the 16S rRNA gene abundance (Table 3). Collectively, our data indicate that the reuterin-producing *L. reuteri* PTA5_F13 was not able to grow in the conditions used for *Campylobacter* growth, but was metabolically active and produced reuterin under microaerophilic culture conditions.

### 3.3. Reuterin Produced by *L. reuteri* PTA5_F13 Kills *C. jejuni* N16-1419 during Co-Cultures

In a next step, we tested the effect of in situ produced reuterin on *Campylobacter* during co-cultures of reuterin-producing *L. reuteri* PTA5_F13 and *C. jejuni* N16-1419 with and without glycerol.

We observed an increase of the 16S rRNA gene copy number indicating the growth of *C. jejuni* N16-1419 when co-cultured with *L. reuteri* PTA8_1 in the presence or without glycerol (Table 3). Similarly, there was no significant difference (*p* > 0.05) in the viable cell counts during incubation with glycerol or without glycerol in the medium. Acetate and succinate were the main metabolites produced, at similar levels to those found in *C. jejuni* N16-1419 mono-cultures. The final pH of the co-cultures with *L. reuteri* PTA8_1 with or without glycerol was similar to the pH of the non-inoculated CAMH media (Table 2).

During co-cultures with reuterin-producing *L. reuteri* PTA5_F13 in CAMH medium with 28 mM glycerol, the viable cell counts of *C. jejuni* N16-1419 drastically decreased below the detection limit (1 log CFU/mL) after 24 h incubation, while the number of 16S rRNA gene copies was reduced by 1.3 ± 0.4 log gene copies (Table 3). Succinate, a main metabolite of *C. jejuni* N16-1419, was low produced, reaching final concentrations of 0.8 ± 0.1 mM compare with 5.3 ± 0.4 mM during mono-cultures. Glycerol was consumed (25.4 ± 0.4 mM, 93%) and acrolein (0.1 ± 0.0 mM), 1,3-PDO (6.2 ± 0.6 mM) and 3-HP (4.9 ± 0.8 mM) were produced, and the pH dropped from 7.3 to 5.8 after 24 h of fermentation, while 2.2 mM lactate was also produced (Table 2). In contrast, in the absence of glycerol, a significant growth *C. jejuni* N16-1419 was observed during co-culture with *L. reuteri* PTA5_F13 (Table 3). This was reflected by a higher metabolite production compared to co-cultures with glycerol, with a higher yield of acetate (7.9 ± 0.5 mM) and succinate (5.7 ± 1.3 mM), while the pH was maintained at 7.3. Altogether, these findings suggest that in the presence of 28 mM glycerol, *L. reuteri* PTA5_F13 synthesised reuterin leading to the killing of *C. jejuni*.

## 4. Discussion

The need for novel strategies to reduce *Campylobacter* in poultry and poultry products becomes more urgent [27]. Biosecurity and hygiene procedures that are implemented to prevent flock colonisation by *Campylobacter* are limited due to uncontrolled environmental factors in organic flocks, and by difficulties to strictly respect biosecurity rules in conventional flocks throughout the rearing stages [28]. Among alternative strategies to prevent *Campylobacter* occurrence in poultry and poultry products, a particular attention has been shifted to biocontrol approaches, involving the use of naturally-produced compounds and microbial competitive exclusion [29].

Reuterin is produced by certain strains of *L. reuteri* during anaerobic fermentation of glycerol and has a broad-spectrum antimicrobial property against enteric pathogens and intestinal bacteria [21]. The combine antimicrobial effect of reuterin and nisin was investigated on a single strain of *C. jejuni* in milk [30]. However, the authors did not provide information on the effective concentration of reuterin, and more specifically on acrolein. Our work reports for the first time the sensitivity of a broad panel of *C. jejuni* and *C. coli* isolates to reuterin and provides a first clear evidence of the killing of *C. jejuni.* The chicken intestine is the main reservoir of *Campylobacter* contaminated meat and human campylobacteriosis [31,32]. Therefore, our data also indicate high activity of reuterin on chicken intestinal strains.

*Campylobacter* spp. exhibited higher sensitivity to reuterin than *E. coli* K12, which is often used for its high susceptibility as an indicator strain for testing reuterin activity [21]. Recently, the antimicrobial activity of reuterin was suggested to involve a reaction of the active compound, acrolein [10] with thiol groups of glutathione or redox-active proteins with subsequent inhibition of redox-base defences causing oxidative stress [12,33]. The addition of exogenous thiol groups was shown to suppress the antimicrobial effect of reuterin [10]. Total intracellular thiol content in *E. coli* cells decreased to about 20% of initial levels after exposure to acrolein [34], and GSH-deficient *E. coli* mutants were significantly more susceptible to acrolein when compared to a wild type strain [33,34]. Genome analysis of several *Campylobacter* strains has suggested that this genus does not encode homologs of the glutathione biosynthesis proteins [35,36] and might, therefore, lack detoxifying capacity against redox modifying compounds such as acrolein. This characteristic likely explains the high sensitivity of *Campylobacter* spp. to reuterin.

The production of reuterin by *L. reuteri* is a strain-specific characteristic [37]. The ability of chicken-derived reuterin-producing *L. reuteri* PTA5_F13 to synthesize reuterin, and inactivate *C.jejuni* in co-culture was evaluated and compared with *L. reuteri* PTA8_1, which does not produce reuterin [17]. The phenotypic similarity of *L. reuteri* PTA8_1 and *L. reuteri* PTA5_F13 was confirmed during mono-cultures without glycerol where there were no significant difference in cell growth and main metabolite (lactate and acetate) production. However, as expected, glycerol was not utilized by *L. reuteri* PTA8_1 during mono- and co-cultures, which lead to no significant difference in the metabolite profile in the presence or absence of glycerol and no reuterin formation. We confirmed reuterin synthesis by chicken-derived *L. reuteri* PTA5_F13 with the formation of acrolein, 1,3-PDO and 3-HP in conditions of the co-cultures, selected to promote the growth of *Campylobacter* spp. The intermediate product of glycerol conversion 3-HPA was not detected, likely because it can be converted to 1,3-PDO in the presence of glucose and also chemically reacts to acrolein at the cultivation conditions used (Figure 1) [10].

Our results show that *C. jejuni* N16-1419 was killed during co-cultures with *L. reuteri* PTA5_F13 in the presence of glycerol. In the absence of glycerol, no anti-*Campylobacter* activity was observed for both reuterin-producing and non-reuterin producing *L. reuteri* strains. Kobierecka et al. [38] reported that oral administration of *L. reuteri* did not significantly reduce the level of *Campylobacter* colonization in chicken. It appears that *L. reuteri PTA5*_F13 adapts to the presence of *C. jejuni* N16-1419 in co-culture by an increase in the production of lactate (2 mM) which significantly reduced the pH of the culture medium to pH 5.8 when glycerol was present. Neal-McKinney et al. [39] showed that CAMH medium with 10 mM lactate or pH 5.12 (by HCl) reduced the growth of *C. jejuni* by 1 log CFU/mL compared to the untreated control. Also, previous in vitro studies have shown that mildly acidic conditions, such pH 5.0 at 42 °C in semisolid Brucella agar [40] and pH 5.5 at 37 °C in biphasic CAMH medium [41] do not affect the growth (plate count and OD600 measurement) of *C. jejuni* after 24 h. Therefore, we assume that the observed drop of pH from 7.3 to 5.8 that occurred during the co-culture with *L. reuteri* PTA5_F13 was not responsible for observed *C. jejuni* inactivation. Our results indicate that reuterin production is the predominant mechanism of *C. jejuni* killing by *L. reuteri* in vitro.

## 5. Conclusions

We identified reuterin as a potent antimicrobial with high activity against a broad panel of *C. jejuni* and *C. coli* isolates. We also showed that in the presence of glycerol, chicken-derived reuterin-producing *L. reuteri* PTA5_F13 has antimicrobial activity against *C. jejuni* in vitro and reuterin production is required for this phenotype. The strong anti-*Campylobacter* effect may have the potential for use for enhancing the decontamination of slaughter-house equipment. Furthermore, a future direction of this research will focus on confirming our in vitro findings in *Campylobacter*-positive chicken gut microbiota, using both in vitro chicken cecum fermentation models and animal experiments.

## Figures and Tables

**Table 1:** Activity of reuterin against a panel of *Campylobacter* spp., expressed in acrolein concentration. A list of tested strains is presented in Table S1.

Organism	Number of Strains	MIC ^a^ (µM)	MBC ^a^ (µM)
*Campylobacter jejuni*	51	1.5–3.0	3.0–5.8
*Campylobacter coli*	20	1.5–3.0	1.5–5.8
*Campylobacter jejuni* ATCC 33560	1	3.0	5.8
*Lactobacillus reuteri*	5	150.0	450.3
*Escherichia coli* K12	1	16.5	50.1

^a^ Minimum inhibitory concentration (MIC) and minimal bactericidal concentrations (MBC) data are calculated from three independent repetitions for each species.

**Table 2:** Glycerol utilisation and metabolite formation of single strain cultures and co-cultures after 24 h incubation with or without glycerol. pH, glycerol, reuterin and metabolite concentrations of single strain and co-cultures with and without glycerol.

Strains	Treatment	pH ^1^	Substrate Consumed	Metabolites Formed					
			Glycerol ^2^ (mM)	Lactate (mM)	Acetate (mM)	Succinate (mM)	Acrolein (mM)	1,3-PDO (mM)	3-HP (mM)
*C. jejuni* N16-1419	Glycerol	7.2 ± 0.1 ^A^	0.0	<0.1 ^A^	4.4 ± 0.5 ^A^	5.3 ± 0.4 ^A^	ND	ND	ND
	No glycerol	7.3 ± 0.1 ^A^	-	<0.1 ^A^	4.0 ± 0.3 ^A^	6.2 ± 0.6 ^A^	ND	ND	ND
*L. reuteri* PTA8-1	Glycerol	6.8 ± 0.1 ^A^	0.0	0.2 ± 0.1 ^A^	1.9 ± 0.1 ^A^	ND	ND	ND	ND
	No glycerol	6.8 ± 0.1 ^A^	-	0.3 ± 0.1 ^A^	2.0 ± 0.1 ^A^	ND	ND	ND	ND
*L. reuteri* PTA5-F13	Glycerol	6.1 ± 0.1 ^A^	12.9 ± 1.1	0.7 ± 0.1 ^A^	3.0 ± 0.1 ^A^	ND	0.2 ± 0.0	5.3 ± 0.3	4.4 ± 0.3
	No glycerol	6.8 ± 0.1 ^B^	-	0.2 ± 0.1 ^B^	1.9 ± 0.1 ^B^	ND	ND	ND	ND
*C. jejuni* N16-1419 + *L. reuteri* PTA8-1	Glycerol	7.2 ± 0.1 ^A^	0.0	<0.1 ^A^	6.8 ± 0.1 ^A^	5.4 ± 0.6 ^A^	ND	ND	ND
	No glycerol	7.3 ± 0.1 ^A^	-	<0.1 ^A^	6.7 ± 0.4 ^A^	5.9 ± 0.8 ^A^	ND	ND	ND
*C. jejuni* N16-1419 + *L. reuteri* PTA5-F13	Glycerol	5.8 ± 0.1 ^A^	25.4 ± 0.4	2.2 ± 0.1^A^	3.4 ± 0.1 ^A^	0.8 ± 0.1 ^A^	0.1 ± 0.0	6.2 ± 0.6	4.9 ± 0.8
	No glycerol	7.3 ± 0.1 ^B^	-	<0.1 ^B^	7.9 ± 0.5 ^B^	5.7 ± 1.3 ^B^	ND	ND	ND

Data presented as the mean and standard deviation of three biological replicates. Mean values with different alphabetical superscript letters are significantly different at *p* < 0.05. ND; not detected with detection limits: 1.0 mM for glycerol, 0.4 mM for succinate, 0.3 mM for 1,3-PDO, 4.4 µM for acrolein and 1.5 mM for 3-HP. ^1^ The pH of the original Cation adjusted Mueller Hinton broth (CAMHB) was 7.3. ^2^ Initial glycerol concentration was 28 mM.

**Table 3:** Viable cell counts and 16S rRNA gene copy number of mono-cultures and co-cultures after 24 h culture in cation adjusted Mueller Hinton (CAMH) medium with or without 28 mM glycerol.

Strains	Viable Cell Counts (Log CFU/mL)		16S rRNA Gene Copy Number (Log Gene copies/mL)	
	Biphasic CAMH with 28 mM glycerol	Biphasic CAMH without glycerol	Biphasic CAMH with 28 mM glycerol	Biphasic CAMH without glycerol
**Mono-cultures**				
*C. jejuni* N16-1419	8.1 ± 0.4 ^A^	8.4 ± 0.6 ^A^	9.6 ± 0.3 ^a^	9.8 ± 0.1 ^a^
*L. reuteri* PTA8_1	7.0 ± 0.2 ^a^	6.6 ± 0.1 ^a^	7.9 ± 0.1 ^A^	7.3 ± 0.4 ^A^
*L. reuteri* PTA5_F13	4.5 ± 0.4 ^A^	6.7 ± 0.1 ^B^	8.0 ± 0.2 ^a^	7.8 ± 0.2 ^a^
Co-cultures *				
*C. jejuni* N16-1419 + *L. reuteri* PTA8_1	7.5 ± 0.4 ^A^	8.5 ± 0.8 ^A^	9.9 ± 0.2 ^a^	10.2 ± 0.1 ^a^
*C. jejuni* N16-1419 + *L. reuteri* PTA5_F13	BDL ^a^	9.3 ± 0.1 ^b^	6.3 ± 0.1 ^A^	10.2 ± 0.1 ^B^

Data presented as the mean and standard deviation of three biological replicates. Mean values with different alphabetical superscript letters are significantly different at *p* < 0.05. * In co-cultures only *C. jejuni* cells were enumerated on selective plates (CampyFood ID Agar) and qPCR BDL, below the detection limit, with a limit of detection 1 log CFU/mL.

## Supplementary Materials

The following are available online at https://www.mdpi.com/2076-2607/8/1/78/s1, Table S1. Antimicrobial resistance profiles of *Campylobacter* spp. of chicken meat, human stool and animal intestines used in this study. CIP, Ciprofloxacin; TET, tetracycline; ERY, erythromycin; MDR, multidrug resistance. Red square, resistant to a specific antibiotic; green squares, susceptible to a specific antibiotic; purple squares, multidrug resistance.
