# Supplementary material for: Reuterin Demonstrates Potent Antimicrobial Activity Against a Broad Panel of Human and Poultry Meat Campylobacter spp. Isolates
Source: Microorganisms. 2020 Jan 6;8(1):78. doi: 10.3390/microorganisms8010078 (PMC7022665; doi:10.3390/microorganisms8010078)
Supplement: Supplementary file 1 [file microorganisms-08-00078-s001.pdf]

Strain ID	Year	Species	Origin	Sample type	Antimicrobial resistance profile			
					CIP (5 µg)	TET (30 µg)	ERY (15 µg)	MDR
N16-1418	2016	*Campylobacter jejuni*	Food	Chicken meat				
N16-1419	2016	*Campylobacter jejuni*	Food	Chicken meat				
N16-1420	2016	*Campylobacter jejuni*	Food	Chicken meat				
N16-1421	2016	*Campylobacter jejuni*	Food	Chicken meat				
N16-1478	2016	*Campylobacter jejuni*	Food	Chicken meat				
N16-1479	2016	*Campylobacter jejuni*	Food	Chicken meat				
N16-1480	2016	*Campylobacter jejuni*	Food	Chicken meat				
N16-1481	2016	*Campylobacter jejuni*	Food	Chicken meat				
N16-2798	2016	*Campylobacter jejuni*	Animal	Intestinal sample				
N16-2801	2016	*Campylobacter jejuni*	Animal	Intestinal sample				
N17-0540	2017	*Campylobacter jejuni*	Food	Chicken meat				
N17-0541	2017	*Campylobacter jejuni*	Food	Chicken meat				
N17-0542	2017	*Campylobacter jejuni*	Food	Chicken meat				
N16-0006	2016	*Campylobacter jejuni*	Human	Stool				
N16-0110	2016	*Campylobacter jejuni*	Human	Stool				
N16-0223	2016	*Campylobacter jejuni*	Human	Stool				
N16-0340	2016	*Campylobacter jejuni*	Human	Stool				
N16-0465	2016	*Campylobacter jejuni*	Human	Stool				
N16-0551	2016	*Campylobacter jejuni*	Human	Stool				
N16-0630	2016	*Campylobacter jejuni*	Human	Stool				
N16-0663	2016	*Campylobacter jejuni*	Human	Stool				
N16-0843	2016	*Campylobacter jejuni*	Human	Stool				
N16-0898	2016	*Campylobacter jejuni*	Human	Stool				
N16-1078	2016	*Campylobacter jejuni*	Human	Stool				
N16-1196	2016	*Campylobacter jejuni*	Human	Stool				
N16-1274	2016	*Campylobacter jejuni*	Human	Stool				
N16-1423	2016	*Campylobacter jejuni*	Human	Stool				
N16-1517	2016	*Campylobacter jejuni*	Human	Stool				
N16-1825	2016	*Campylobacter jejuni*	Human	Stool				
N16-2032	2016	*Campylobacter jejuni*	Human	Stool				
N16-2239	2016	*Campylobacter jejuni*	Human	Stool				
N16-2378	2016	*Campylobacter jejuni*	Human	Stool				
N16-2527	2016	*Campylobacter jejuni*	Human	Stool				
N16-2587	2016	*Campylobacter jejuni*	Human	Stool				
N16-2776	2016	*Campylobacter jejuni*	Human	Stool				
N16-2860	2016	*Campylobacter jejuni*	Human	Stool				
N16-2952	2016	*Campylobacter jejuni*	Human	Stool				
N17-0061	2017	*Campylobacter jejuni*	Human	Stool				
N17-0146	2017	*Campylobacter jejuni*	Human	Stool				
N17-0204	2017	*Campylobacter jejuni*	Human	Stool				
N17-0266	2017	*Campylobacter jejuni*	Human	Stool				
N17-0338	2017	*Campylobacter jejuni*	Human	Stool				
N17-0459	2017	*Campylobacter jejuni*	Human	Stool				
N17-0515	2017	*Campylobacter jejuni*	Human	Stool				
N17-0632	2017	*Campylobacter jejuni*	Human	Stool				
N17-0726	2017	*Campylobacter jejuni*	Human	Stool				
N17-0828	2017	*Campylobacter jejuni*	Human	Stool				
N17-0916	2017	*Campylobacter jejuni*	Human	Stool				
N17-1066	2017	*Campylobacter jejuni*	Human	Stool				
N17-1110	2017	*Campylobacter jejuni*	Human	Stool				
N17-1425	2017	*Campylobacter jejuni*	Human	Stool				
N16-0075	2016	*Campylobacter coli*	Human	Stool				
N16-0086	2016	*Campylobacter coli*	Human	Stool				
N16-0224	2016	*Campylobacter coli*	Human	Stool				
N16-0322	2016	*Campylobacter coli*	Human	Stool				
N16-0431	2016	*Campylobacter coli*	Human	Stool				
N16-0444	2016	*Campylobacter coli*	Human	Stool				
N16-0649	2016	*Campylobacter coli*	Human	Stool				
N16-0754	2016	*Campylobacter coli*	Human	Stool				
N16-0825	2016	*Campylobacter coli*	Human	Stool				
N16-0993	2016	*Campylobacter coli*	Human	Stool				
N16-0825	2016	*Campylobacter coli*	Human	Stool				
N16-0993	2016	*Campylobacter coli*	Human	Stool				
N16-2189	2016	*Campylobacter coli*	Food	Chicken meat				
N16-2190	2016	*Campylobacter coli*	Food	Chicken meat				
N16-2726	2016	*Campylobacter coli*	Food	Chicken meat				
N17-0679	2017	*Campylobacter coli*	Food	Chicken meat				
N16-2189	2016	*Campylobacter coli*	Food	Chicken meat				
N16-2190	2016	*Campylobacter coli*	Food	Chicken meat				
N16-2726	2016	*Campylobacter coli*	Food	Chicken meat				
N17-0679	2017	*Campylobacter coli*	Food	Chicken meat				
